# Estimating selection on the act of inbreeding in a population with strong inbreeding depression

**DOI:** 10.1111/jeb.13376

**Published:** 2018-10-16

**Authors:** Eva Troianou, Jisca Huisman, Josephine M. Pemberton, Craig A. Walling

**Affiliations:** ^1^ Institute of Evolutionary Biology School of Biological Sciences University of Edinburgh Edinburgh UK

**Keywords:** genomics, inbreeding, inbreeding avoidance, natural selection, quantitative genetics

## Abstract

Inbreeding depression is widely regarded as a driving force in the evolution of dispersal, mate choice and sperm selection. However, due to likely costs of inbreeding avoidance, which are poorly understood, it is unclear to what extent selection to avoid inbreeding is expected in nature. Moreover, there are currently very few empirical estimates of the strength of selection against the act of inbreeding (mating with a relative), as opposed to the fitness costs of being inbred. Here, we use data from the individual‐based study of red deer on the Scottish island of Rum, a strongly polygynous system which harbours a large inbreeding load, to estimate selection against the act of inbreeding for each sex. We use pedigree and genomic estimates of relatedness between individuals and measure fitness using both lifetime breeding success (number of calves born) and lifetime reproductive success (number of calves surviving to independence), with the latter incorporating inbreeding depression in calf survival. We find for both sexes that the repeatability of the act of inbreeding was low (< 0.1), suggesting little among‐individual variation for this trait on which selection can act. Using the genomic measures, there was significant selection against the act of inbreeding in males, but not in females, and there was considerable uncertainty in the estimate in both sexes. We discuss possible explanations for these patterns and their implications for understanding the evolution of inbreeding avoidance in natural populations.

## Introduction

Inbreeding is the mating of individuals related by ancestry. Offspring produced by such a mating often suffer from a reduction in fitness compared to offspring produced by unrelated parents, known as inbreeding depression. Inbreeding and its deleterious consequences are topics of great interest in many fields within biology. In conservation, inbreeding depression raises concerns about population persistence (Keller & Waller, 2002), whereas in agriculture it has an impact on economic profitability (Leroy, 2014). In evolutionary biology, its detrimental consequences are considered to have shaped the evolution of aspects of breeding systems in plants (Charlesworth & Charlesworth, 1987; Charlesworth, 2006) and animals (Blouin & Blouin, 1988; Pusey & Wolf, 1996).

### The inbreeding paradox

An important question in understanding the evolutionary consequences of inbreeding is how the propensity to inbreed has itself evolved. Answering this question requires the investigation of inbreeding strategies in a range of systems, as well as the quantification of key parameters that cause evolutionary change. It is often assumed that inbreeding depression has driven the evolution of mechanisms that result in inbreeding avoidance, such as dispersal, choice among related or unrelated mates and sperm selection (Blouin & Blouin, 1988; Pusey & Wolf, 1996). However, inbreeding avoidance is not always observed in nature, as many plants and hermaphroditic animals display a wide range of self‐fertilization rates, the most intensive form of inbreeding (Goodwillie *et al*., 2005; Jarne & Auld, 2006). Additionally, there are an increasing number of studies investigating the inbreeding behaviour or strategy of experimental and wild animal populations with biparental inbreeding. Diverse patterns of inbreeding strategy have been reported (Szulkin *et al*., 2013), with several studies not detecting inbreeding avoidance (e.g. Keller & Arcese, 1998; Jennions *et al*., 2004; Hansson *et al*., 2007; Szulkin *et al*., 2009; Rioux‐Paquette *et al*., 2010; Ala‐Honkola *et al*., 2011; Reid *et al*., 2015b,c) even in populations in which strong inbreeding depression is present, pointing towards an ‘inbreeding paradox’.

### The evolution of inbreeding strategies

There are a number of possible explanations for the observation that inbreeding is not always avoided. There may be costs of inbreeding avoidance, for example, costs of foregoing a mating or costs of dispersal, and there may be potential benefits of inbreeding, for example through kin selection (Smith, 1979; Waser *et al*., 1986; Kokko & Ots, 2006; Puurtinen, 2011). An increasing number of theoretical investigations suggest that understanding the evolution of inbreeding avoidance in biparental species requires far more than just estimating inbreeding depression (Szulkin *et al*., 2013) and that the conditions under which inbreeding avoidance is likely to evolve are more restrictive than previously thought. Three theoretical clarifications are of relevance here. First, any kin‐selected benefits depend critically on which sex is mate‐limited and which sex provides parental care. In polygynous systems with female‐only parental care, males are typically mate‐limited whereas females are resource‐limited. For a male under these conditions and from a kin selection perspective, mating with a related female is of equal value to mating with an unrelated female: the shared alleles in the related female are passed on to the next generation regardless of his choice (she will mate anyway), as are his own alleles if he manages to mate. The only effect of his choice is whether his and her allele copies end up in the same offspring, or in different offspring, and this is irrelevant to the male if he does not provide care (Waser *et al*., 1986; Duthie & Reid, 2016). Males in such systems are therefore expected to avoid or tolerate inbreeding but never to actively pursue inbreeding (Waser *et al*., 1986; Duthie & Reid, 2016). Females, on the other hand, could accrue inclusive fitness benefits by providing male relatives with additional reproductive success – if she had not mated with him, he likely would have sired one less offspring, and fewer of their shared alleles would be passed on to the next generation. However, any inbred offspring a female produces is at the expense of an outbred offspring, the opportunity cost of inbreeding (Waser *et al*., 1986). Thus, the sexes may experience different selection regimes with respect to inbreeding and may have conflicting interests, but the exact nature of any differences is dependent on details of the life history, such as the amount of parental care and the intensity of competition for mates.

Second, simulations indicate that the population sizes under which biparental inbreeding avoidance or preference can evolve are relatively restrictive (Duthie & Reid, 2016). It is in small, viscous populations that inbreeding is most likely to occur, potentially generating selection for inbreeding avoidance or preference. However, in small populations selection is relatively inefficient, reducing the chances of such strategies evolving. In contrast, in large populations, selection is efficient but inbreeding is likely to be rare, generating little selection to avoid inbreeding.

Third, inbreeding and inbreeding depression occur in different generations; consequently, the costs for an individual that inbreeds cannot be assessed only from the reduction in the fitness of its offspring (Reid *et al*., 2015a). From a quantitative genetic perspective, fitness is best defined as the number of zygotes produced by a zygote (Arnold, 1985). Following this definition, it is possible for inbreeding to reduce fitness if it causes gamete incompatibility or embryo inviability (Reid *et al*., 2015a). However, avoiding inbreeding may also reduce the number of zygotes produced if costs of dispersal, loss of breeding opportunities or energy costs of locating unrelated individuals exist. A different measure of fitness, incorporating survival of zygotes until maturity, can give insight into whether foregoing the costs of avoiding inbreeding outweighs the cost of inbreeding depression in the offspring.

### Quantifying the costs of inbreeding

Most studies in wild animal populations quantify the costs of inbreeding as inbreeding depression, by measuring the effect of an individual's coefficient of inbreeding depression on its own fitness and therefore measuring ‘selection against being inbred’ (e.g. see Keller & Waller, 2002; Kruuk *et al*., 2002; Pemberton *et al*., 2017). However, in the light of the above, selection on inbreeding should be assessed from the perspective of the individual who mates with a relative or not (Reid *et al*., 2015a). This can be achieved by quantifying the relationship between an individual's relatedness to its mate and its own fitness and thus estimating ‘selection on the act of inbreeding’. Furthermore, comparing the magnitude of selection against being inbred and against the act of inbreeding might explain why inbreeding is observed even in populations where strong inbreeding depression is present. When there is negative selection on being inbred, but zero or positive selection on the act of inbreeding, this would imply that there are certain benefits associated with inbreeding or costs associated with its avoidance. Consequently, inbreeding avoidance would not always be the optimal inbreeding strategy.

### Pedigree and genomic estimators of inbreeding and relatedness

Investigating inbreeding and its consequences requires precise quantification of the genetic structure of a population. Pedigree reconstruction is required to infer relationships among individuals (Pemberton, 2008; Szulkin *et al*., 2013). However, pedigree reconstruction can be a slow and sometimes difficult task for wild populations because it requires data from multiple generations and often requires genetic support. Depending on methodology and sampling completeness, parentage assignment may be unsuccessful (no parent assigned) or contain errors (wrong parent assigned). Missing pedigree links introduce downward bias to the estimation of inbreeding and pairwise relatedness, because unidentified parents are assumed to be unrelated to the other members of the population, whereas wrong pedigree links will cause error. For example, when a social pedigree was corrected using genetic markers, estimates of inbreeding depression in most traits increased in the song sparrows (*Melospiza melodia*) of Mandarte Island (Reid *et al*., 2014).

The restrictions that arise from pedigrees have led to estimation of individual inbreeding and pairwise relatedness directly from molecular data (David, 1998; Queller & Goodnight, 2012). Initially, small panels of genetic markers, such as microsatellites, were available for natural populations but did not prove to be very precise, since microsatellite homozygosity is often only weakly correlated with identity by descent (IBD) (Balloux *et al*., 2004; Slate *et al*., 2004; Csilléry *et al*., 2006). However, with the recent availability of high‐density panels of single nucleotide polymorphism (SNP) data, marker‐based measures provide a more accurate estimate of genomewide homozygosity (Hoffman *et al*., 2014; Kardos *et al*., 2015). Additionally, these high‐density panels can capture variation in identity by descent introduced by chromosome assortment and recombination, which cannot be captured by even a perfect pedigree and therefore may increase power to detect inbreeding (Visscher *et al*., 2006; Hill & Weir, 2011).

### The red deer

Here, we consider selection on the degree of biparental inbreeding in a wild mammal population. We use a population of red deer (*Cervus elaphus*) that has been under intense monitoring for more than 40 years (Clutton‐Brock *et al*., 1982). Substantial levels of inbreeding have been detected in this study population. In a pedigree analysis, out of 821 calves born between 1980 and 2010 for which all grandparents were known, 42% had nonzero pedigree inbreeding coefficients (Walling *et al*., 2011). In common with many species with biparental inbreeding, only a few individuals are highly inbred, and many are slightly inbred (see Fig. S2). Inbreeding depression is also evident in the population with both juvenile and adult traits affected (Walling *et al*., 2011; Huisman *et al*., 2016). For example, a previous study found a decline in lifetime breeding success for individuals produced from a half‐sib mating of 72% for females and 95% for males (Huisman *et al*., 2016).

The red deer is a strongly polygynous species. Females are distributed in loose matrilineal groups, and during the breeding season, known as the rut, males compete to defend female groups and mate with those females in their harem which come into oestrus (Clutton‐Brock *et al*., 1982). The most successful males hold stable harems of ten or more females over several consecutive days, whereas unsuccessful males may hold a single female for the occasional day or none at all. Behavioural oestrus is brief (often only an hour or so), mating typically occurs only once or twice per oestrus, and it is rare for a male to have more than one oestrous female in his harem at one time, suggesting there are low opportunity costs for a male that mates with a relative. Whereas females have zero or one calf per year throughout adult life, males often sire multiple calves per year, but over shorter windows of years, typically between the ages of seven and 11 (Nussey *et al*., 2009). Just 9% of matings are between pairs that have mated before (Stopher *et al*., 2012). Thus, individuals of both sexes have multiple offspring by different mates over their lifetimes (albeit over different timescales) making the degree of biparental inbreeding a repeated measures trait. Overall, there is more inbreeding in the population than expected under either random mating or more realistic simulations capturing temporal and spatial aspects of each rut (Stopher *et al*., 2012).

In traits measured repeatedly on the same individual, it is common to partition the phenotypic variance of the trait into a within‐individual and a between‐individual component of variance (Sokal & Rohlfe, 1981; Lessells & Boag, 1987; Boake, 1989). This allows estimation of the proportion of the phenotypic variance which arises from permanent differences between individuals, both genetic and environmental. This permanent or repeatable component of the relatedness between an individual and its mate can be thought of as the propensity of an individual to mate with a relative. This can then be correlated with measures of fitness in order to estimate selection on this propensity. In the case of red deer, we therefore need to estimate the repeatability of the act of mating with a relative in order to determine whether there is variation among individuals in this trait upon which selection can act. The repeatability is generally thought to represent an upper limit to the heritability, such that traits with low repeatability will have low heritability and thus limited ability to respond to selection (Boake, 1989; Lynch & Walsh, 1998), although this may not always be the case (Dohm, 2002).

Here, we investigate, for both sexes, whether relatedness to a mate is repeatable and jointly estimate selection on the degree to which individuals mated with a relative and on being inbred. This allowed us to investigate whether there may be any benefits associated with the act of inbreeding even though inbreeding depression is present in the population. We used both pedigree and genomic estimators of inbreeding and relatedness in order to assess the performance of each method. Since we found evidence for selection against relatedness to mate in males, we explored possible proximate mechanisms for this selection by regressing male traits associated with lifetime breeding success on relatedness to mate and by comparing relatedness to mate with relatedness to harem members.

## Materials and methods

### Study population and data collection

The study population of red deer inhabits the North Block of the Isle of Rum, Inner Hebrides, Scotland (57^o^03′N, 06^o^21′W) (Clutton‐Brock *et al*., 1982). The population has been intensively monitored since 1972, and the culling of individuals in the study area has been suspended since 1973. The study area is censused weekly, and individuals are recognized from natural markings or artificial tags. The censuses become daily during the calving season (May–July) and the rut (September–November) seasons. Approximately 80% of calves are caught during the calving season, and they are weighed and artificially marked. Since 1982, an ear punch and blood sample have been taken from each individual for DNA analysis. Some individuals have also been sampled when tranquillized, from cast antlers or *post‐mortem*. Here, we use data from matings between 1970 and 2014, resulting in calves born between 1971 and 2015.

### Pedigree measures

Pedigree coefficients of relatedness to mates (*R*_PED_) and inbreeding (*F*_PED_) were obtained from a multigenerational pedigree. Only individuals for which both parents were known were included. In these kinds of analyses, there is a trade‐off between the amount of pedigree information available to estimate relatedness and inbreeding coefficients, and sample size (Huisman *et al*., 2016). In our study population, a minimum restriction of both parents known enables substantial sample sizes while accepting that some relatedness and thus inbreeding goes undetected. The pedigree reconstruction was accomplished by a combination of genetic and behavioural data using a likelihood‐based approach (see Huisman *et al*., 2016; Huisman, 2017 for details). Wright's inbreeding coefficients and coefficients of relatedness were calculated for all individuals in the R‐package Pedantics (Morrissey & Wilson, 2010). An individual's pedigree inbreeding coefficient measures the probability of two alleles at any locus being identical by descent, whereas the coefficient of relatedness measures the expected proportion of alleles shared by two individuals that are identical by descent.

### Genomic measures

Genomic relatedness to mates (*R*_GRM_) and inbreeding coefficients (*F*_GRM_) were calculated using 37 410 autosomal SNPs in the software GCTA (Yang *et al*., 2011) (see Huisman *et al*., 2016 for details). *R*_GRM_ between individuals *j* and *k* was estimated by the following:

$$R_{\mathrm{GRM}}=\frac{1}{N}\sum_{i=1}^{N}\frac{\left(x_{\mathrm{ij}}-2p_{i}\right)\left(x_{\mathrm{ik}}-2p_{i}\right)}{2p_{i}\left(1-p_{i}\right)}$$

where *p*_*i*_ is the allele frequency at locus *i*, and *x*_ij_ and *x*_jk_ are the number of copies of the reference allele (0, 1, 2) for individual *j* and *k*, respectively. *F*_GRM_ for each individual was the inbreeding estimator ${\hat{F}}^{\mathrm{III}}$ in (Yang *et al*., 2011) and calculated as follows:

$$F_{\mathrm{GRM}}=\frac{1}{N}\sum_{i}^{N}\frac{x_{i}^{2}-\left(1+2p_{i}\right)x_{i}+2p_{i}^{2}}{2p_{i}\left(1-p_{i}\right)}$$

where *p*_*i*_ is the allele frequency at locus *i*, and *x*_*i*_ is the number of copies of the reference allele (0, 1, or 2). These two variables estimate the similarity of gametes between pairs of individuals in an individual's genome or relative to a random sample from the population, they are centred on zero and unrelated pairs, and outbred individuals take values below zero (Yang *et al*., 2011). Figures S1 and S2 show distributions and comparisons of pedigree and genomic *R* and *F* in our data set.

### Fitness measures

First, fitness was measured as lifetime breeding success (LBS), defined as the total number of offspring produced over an individual's lifetime. Second, fitness was also measured as lifetime reproductive success (LRS), defined as the number of offspring produced by an individual that reached independence, that is the age of 2 years in red deer, capturing variation in offspring survival due to inbreeding depression.

Lifetime breeding success was calculated for individuals who were known to have died a natural death, thus excluding those who were culled when ranging outside the study area. Individuals who were still alive in 2016 and born at least 14 years (for females) or 12 years (for males) before 2016 were also included, because only 1.9% of pregnancies occur in females older than 15 and only 2.5% of the calves are sired by males over the age of 13 (Huisman *et al*., 2016). LRS was calculated for individuals for which LBS was known or were born 14 + 2 years (for females) or 12 + 2 years (for males) before 2016.

Lifetime allelic fitness (LAF), defined as the total number of identical by descent copies contributed from a focal individual to the next generation (Reid *et al*., 2015a), has recently been used as a measure of fitness in studies of the evolution of inbreeding avoidance, especially in the presence of potential kin selective benefits. As discussed in the introduction, this only applies to females in polygynous species such as red deer. However, when we explored estimates of lifetime allelic fitness (LAF) in the deer study system, we found they are very strongly correlated with LBS (e.g. *r *=* *0.994 in females and *r* = 0.995 in males for the genomic measures). This implies that kin selection benefits to females cannot play an important role in this system, and given that it does not provide any additional information, we do not consider this measure further here.

### Standardization of variables

All response and predictor variables were standardized prior to analysis to allow comparison across studies. Standardizations were performed within the subsets of data used for each model (sample sizes for these models are given in Table S1). Note that the data are restricted to individuals that had at least one offspring over their lifetime, because without an offspring it is not possible to calculate the relatedness between two mates in species like red deer that do not form a pair bond. Relative fitness (*w*) for each individual was calculated by dividing an individual's absolute fitness by the mean absolute fitness of the individuals in each data subset. Pedigree and genomic coefficients of relatedness to mates and inbreeding coefficients were standardized to have a mean of 0 and a standard deviation of 1, by subtracting the mean and dividing by the standard deviation of each subset of individuals.

### Repeatability

Repeatability was estimated by fitting linear mixed models with either *R*_PED_ or *R*_GRM_ as a response variable and an individual's identity as a random effect (Wilson *et al*., 2010). This allowed the variance in the response variable to be partitioned into among‐ and within‐individual components, with the among‐individual component representing repeatability. The significance of the repeatability was tested using log‐likelihood ratio tests (LRTs) comparing models with and without the random effect of individual identity. The test statistic was calculated as twice the difference of the log‐likelihoods between the full and the reduced model, and it was compared to a chi‐squared distribution with one degree of freedom in order to obtain the *P*‐value. We also fitted models that included both sexes, and constrained parameters of interest to be equal to test for differences between the sexes.

### Selection gradients

We estimated selection as the association between the repeatable component of relatedness to a mate and relative fitness. In order to estimate selection on the act of inbreeding and selection on being inbred simultaneously (Lande & Arnold, 1983), we ran multivariate models with relative fitness, *R*_PED_ and *F*_PED_, or with relative fitness, *R*_GRM_ and *F*_GRM_ as response variables and an individual's identity as a random effect, for each sex separately. In these models, the residual variance for relative fitness and *R*_GRM_ or *R*_PED_ was fixed to zero, forcing these variances to be estimated as among‐individual components. This allows the estimation of the covariance between relative fitness and the repeatable component of relatedness to a mate as well as the covariance between relative fitness and an individual's own inbreeding coefficient (see Morrissey *et al*., 2012 and Walling *et al*., 2014 for details on these models). By using an antedependence structure, this covariance matrix can be reparameterized in terms of two variances and also a regression coefficient that in this context is a selection gradient (Butler *et al*., 2009) (see also SI of Thomson *et al*., 2017). Selection gradients were therefore directly estimated from these models as the regression of fitness on the repeatable component of relatedness to a mate and the regression of fitness on an individual's own inbreeding coefficient. This allows estimation of selection on relatedness to a mate independent of the effect of an individual's own inbreeding coefficient on fitness. Significance was calculated using LRTs comparing models with the parameter of interest estimated vs. fixed at zero, as above. In order to visualize these relationships, we extracted the best linear unbiased prediction (BLUP) of an individual's propensity to mate with a relative from the ASReml‐R models and plotted these against standardized measures of fitness. BLUPs represent model predictions and are estimated with error; however, they are used here for visualization purposes only and it is important to note that parameter estimates are not based on analyses of these BLUP values (Hadfield *et al*., 2010). Analyses were performed in R version 3.2.0 (R Core Team 2015) with the packages lme4 (Bates *et al*., 2015) and ASReml‐R (Butler *et al*., 2009).

### Outlier analysis

Immigrant males to the population are thought to be particularly successful at siring offspring and relatively unrelated to the rest of the population (Huisman *et al*., 2016). We therefore checked whether any patterns found were driven by a few individuals of either sex who were genetically dissimilar (very negative *R*_GRM_ or low *R*_PED_) from all potential mates. To do this, we calculated for each individual the average relatedness to all potential mates, that is all opposite‐sex individuals that had offspring in the years he/she had offspring, and repeated analyses removing any individuals with values more than four standard deviations from the mean.

### Proximate explanations for selection against the act of inbreeding in males

We tested whether certain male traits that are known to predict male LBS, specifically age, antler weight (from cast antlers identified to their owner) and cumulative female days held (a behavioural measure of success from daily rut censuses), were associated with mean *R*_GRM_, with the prediction that the association should be negative if these associations explain the observed selection on *R*_GRM_. In each case, we ran a linear mixed model with *R*_GRM_ as the response variable and a random effect of male ID to account for the repeated measures. In the case of age, there are 11 cases in which males rutted sufficiently long, and a daughter matured sufficiently fast, that a father–daughter mating occurred, so we also tested whether the age relationship changed when these cases were omitted. Finally, the mechanism for selection against relatedness to mate could be that successful males have fewer relatives in their harem or that successful males are less likely to mate with more closely related females within their harem. We tested these possibilities with linear mixed models with *R*_GRM_ as the response variable and male LBS as a fixed effect to test the first hypothesis. We then added an interaction between male LBS and whether or not the pair mated as a fixed effect to test whether relatedness to harem members varied less strongly with LBS than relatedness to mates, which would indicate that males with high LBS avoid mating with relatives within their harem (see Fig. S4 for a visual representation of these predictions). These analyses were conducted on data collected since 1972 (rather than 1970 as above), because this is when harem membership records started. All models included male ID as a random effect and used nonstandardized data.

## Results

### Repeatability of relatedness to mate

**Figure 1:**
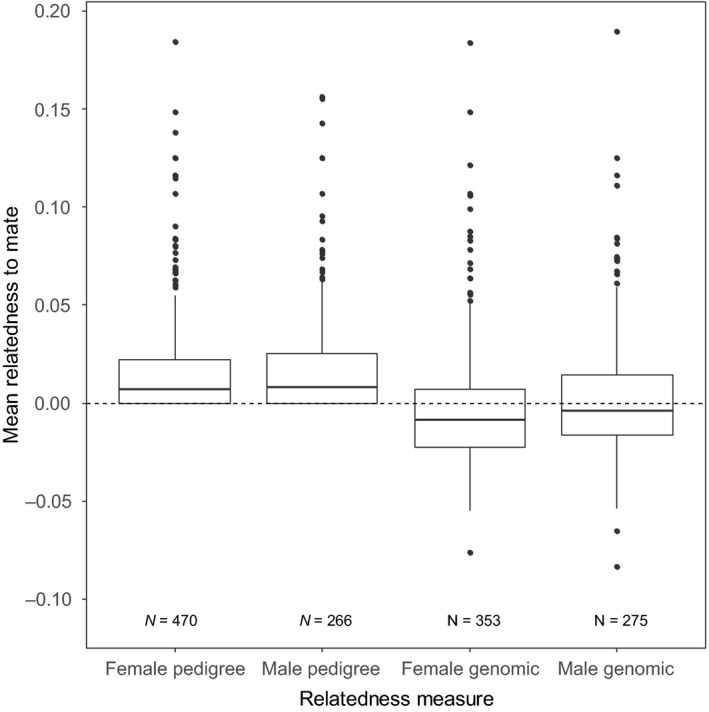
Distributions of individuals’ average relatedness to mate, for those with at least two mates (i.e. for which an average is not based on a single value). Horizontal bar = median; box = 25%–75%; vertical line = ±1.5 × interquartile range above or below the 25th or 75th percentile; points are individual outliers from this range. Numbers below bars give the number of individuals, N. For distributions of relatedness to each mate, see Fig. S2, and for average and standard deviation, see Table S1

**Figure 2:**
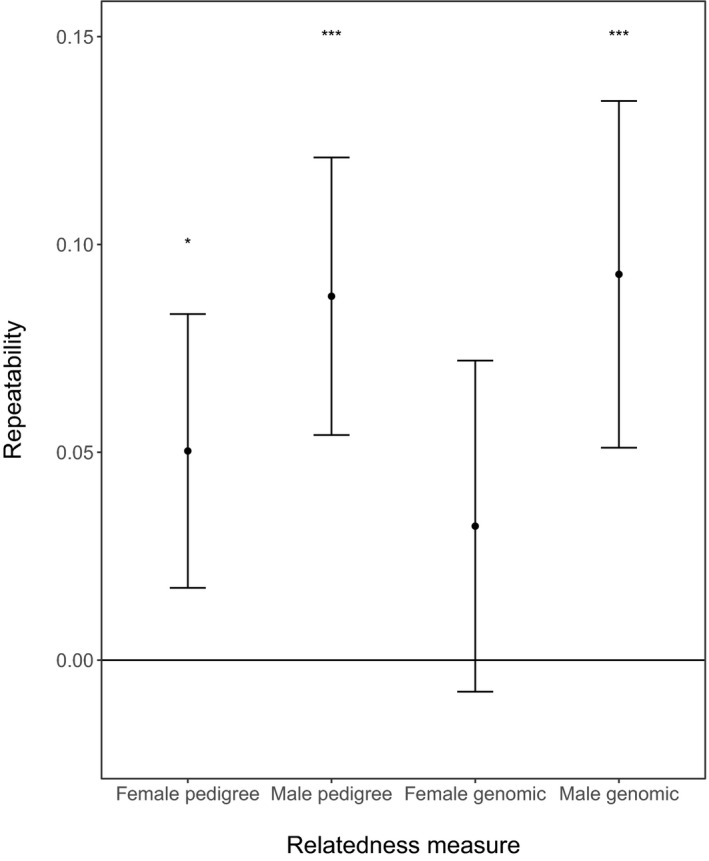
Estimated repeatability of relatedness to mates for males and females from pedigree and genomic estimates; error bars indicate 95% confidence intervals and asterisks standard significance levels

There was considerable variation in the relatedness to mates among both females and males (Fig. 1, Table S1). However, relatively little of this variation was repeatable among individuals. In females, repeatability was significant when using the pedigree estimate of relatedness to a mate (*r* = 0.0503 ± 0.0168, *P* < 0.001, Fig. 2, Table S2), but not when using the genomic estimate (*r* = 0.0322 ± 0.0203, *P* = 0.0929). In males, repeatability was significant when using both estimates (pedigree: *r* = 0.0875 ± 0.0170, *P* < 0.001; genomic: *r* = 0.0968 ± 0.0213, *P* < 0.001, Fig. 2). Males were significantly more repeatable in their propensity to mate with a relative than females when using both pedigree (*χ* ^2^ = 6.28, *P* = 0.0122) and genomic measures (*χ* ^2^ = 12.6, *P* < 0.001). However, repeatability was low (< 0.1) in all cases, suggesting little variation in the propensity of individuals to mate with a relative in either sex. The finding of significant repeatability in *R*_PED_ but not in *R*_GRM_ in females might be due to the fact that an *R*_PED_ of zero was assigned to a large proportion of matings (Fig. S1), which will inflate the repeatability estimate, but these zeroes most likely reflect pedigree incompleteness rather than true (un)relatedness.

### Correlations between fitness measures

In females, the number of offspring surviving to independence (LRS) ranged from zero to nine and was only moderately correlated with the number of offspring born (LBS) *(r *=* *0.67, *t*_344_ = 16.9, *P* < 0.0001; Fig. S3). The correlation in males was much higher (*r *=* *0.87, *t*_231_ = 27.0, *P* < 0.0001; Fig. S2). This difference between the sexes arises because females provide extensive maternal care and vary in their success in rearing calves to independence. Males provide no parental care and mate seemingly at random with respect to maternal success, so LRS more closely reflects LBS.

### Selection gradients

Although the repeatability analyses presented above suggest little variation in the propensity to mate with a relative, especially in females, the point estimate of the strength of selection on this trait may still provide interesting insights.

#### Females

**Figure 3:**
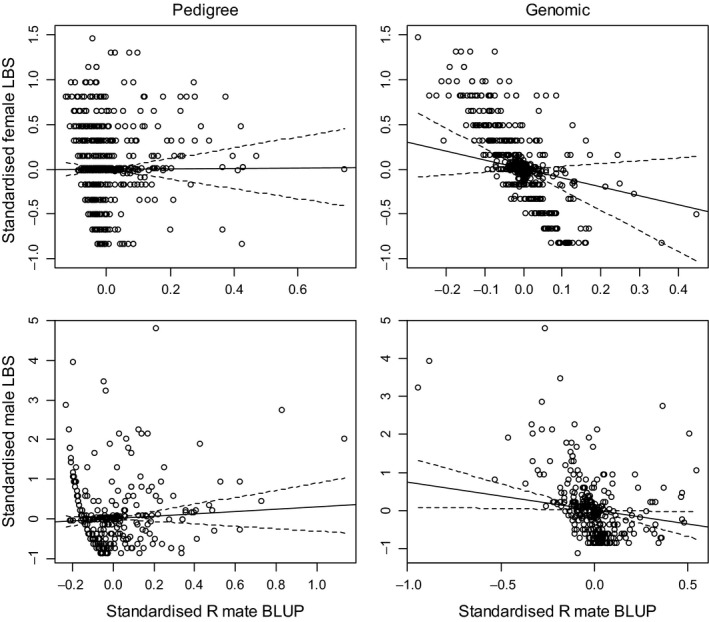
Graphical representation of selection gradients (solid lines) and their 95% confidence intervals (dashed lines). Points show best linear unbiased predictions (BLUPs) for an individual's repeatable value of relatedness to mate (R mate BLUP) and their lifetime breeding success (LBS) on the standardized scale. Note that this is for illustrative purposes only and the statistical analyses did not use BLUPS and instead used all observations for each individual

No association was found between pedigree relatedness to mates (*R*_PED_) or inbreeding (*F*_PED_) and relative LBS (Table 1; Fig. 3), although the sign for inbreeding is consistent with inbreeding depression. The association between *R*_PED_ and LRS was negative and significant (*β *= −0.930 ± 0.509, *P* = 0.0426, Table 1) suggesting that females who tended to mate with relatives produced fewer calves that survived to independence. The association between *F*_PED_ and LRS was not significant, but the sign was again consistent with inbreeding depression.

**Table 1:** Standardized selection gradients (*β*) for relatedness to mate (*R*_PED_ or *R*_GRM_) and being inbred (*F*_PED_ or *F*_GRM_) with standard errors (SE), Chi square and P values (significant terms are in bold)

Sex	Relatedness and inbreeding	Fitness measure	Relatedness				Inbreeding			
			*β*	SE	*χ* ^2^	*P*	*β*	SE	*χ* ^2^	*P*
Female	Pedigree	LBS	0.0271	0.292	0.000705	0.933	−0.0149	0.0294	0.251	0.616
		LRS	**−0.930**	**0.509**	**4.11**	**0.0426**	−0.0598	0.0526	1.22	0.270
	Genomic	LBS	−0.991	0.668	3.52	0.0606	−0.0666	0.0351	2.36	0.125
		LRS	**−2.40**	**1.47**	**8.50**	**0.0035**	−0.127	0.070	2.01	0.157
Male	Pedigree	LBS	0.302	0.310	1.09	0.296	−0.136	0.084	2.18	0.140
		LRS	−0.135	0.372	0.154	0.695	−0.201	0.109	2.20	0.138
	Genomic	LBS	**−0.743**	**0.334**	**6.38**	**0.0115**	**−0.283**	**0.061**	**19.1**	**< 0.001**
		LRS	**−1.10**	**0.43**	**9.25**	**0.0024**	**−0.334**	**0.076**	**16.8**	**< 0.001**
	Genomic^a^	LBS	**−0.716**	**0.383**	**4.63**	**0.031**	**−0.274**	**0.061**	**18.7**	**< 0.001**
		LRS	**−**0.753	0.444	3.78	0.052	**−0.288**	**0.071**	**15.2**	**< 0.001**

a Excluding one outlier male.

The association between the genomic measure *R*_GRM_ and LBS was negative but not significant (*β *= −0.991 ± 0.668, *P* = 0.0606; Table 1; Fig. 3), whereas the association between *R*_GRM_ and LRS was negative and significant (*β *= −2.40 ± 1.47, *P* = 0.0035; Table 1). Thus, females who were more likely to mate with a relative produced fewer calves that survived to independence, partly due to a nonsignificant trend for them to give birth to fewer calves, but largely due to lower offspring survival. The associations between *F*_GRM_ and LBS and LRS were both negative, indicative of inbreeding depression in female fecundity and rearing success, as demonstrated elsewhere (Huisman *et al*., 2016), but did not reach statistical significance in this data set. This may be a result of individuals with LBS of zero being excluded in the current analysis (see Methods).

#### Males

We found no significant association between a males’ pedigree relatedness to his mates and his relative LBS or LRS (Table 1; Fig. 3). There was a tendency for males with higher *F*_PED_ to have lower relative fitness, consistent with inbreeding depression, but this effect was not significant in this data set (Table 1).

In contrast, when using genomic measures of relatedness, we found significant selection against mating with a relative (*β *= −0.743 ± 0.334, *P* = 0.0115; Table 1; Fig. 3). As expected, estimates were larger when using relative LRS rather than relative LBS as the measure of fitness (*β *= −1.10 ± 0.43, *P* = 0.0024; Table 1), because LRS incorporates the effect of inbreeding depression in the offspring of the focal individual. In addition, using the genomic estimator *F*_GRM_ there was significant inbreeding depression in males measured using both LBS and LRS (*β *= −0.283 ± 0.061, *P* < 0.001 and *β *= −0.334 ± 0.076, *P* < 0.001, respectively; Table 1).

The outlier analysis identified a single male who was least related to his potential mates (his mean *R*_GRM_ = −0.054, compared to −0.024 to 0.040 (min‐max) for all other males, Fig. S5). The point estimates for the selection gradients were lower and less significant when excluding this single outlier male (Table 1), but did not qualitatively alter our results. From Fig. 3, there appear to be two males with extreme negative BLUP values for *R*_GRM_. Removing these males reduced the estimated selection gradient slightly and increased the error in the estimate and thus reduced the significance (*β *= −0.523 ± 0.461, *χ* ^2^ = 1.66 *P* = 0.198). For females, one clear outlier produced a single inbred calf (Fig. S5). Removal of this female reduced the estimated selection gradient slightly, and further reduced the significance of this value (*β *= −0.803 ± 0.678, *χ* ^2^ = 1.77, *P* = 0.184), but again did not qualitatively alter our results.

### Proximate explanations for selection against the act of inbreeding in males

Age was known for all males with known LBS. There was a positive association between male age and relatedness to mate (linear model with *R*_GRM_ as response variable, *β* = 0.0031 ± 0.0009, *t*_1446_ = 3.63, *P* = 0.0003), but this disappeared when father–daughter matings were excluded (−1.217E‐4 ± 6.902E‐4, *t*_1435_ = −0.176, *P* = 0.88). Antler weight was known for 247 male‐years in the data set and unknown for 507. There was no association between antler weight and *R*_GRM_ (*β *= −2.633E‐5 ± 1.594E‐5, *t*_588_ = −1.652, *P* = 0.0991, adj. *R* ^2^ = −0.002926). Female days held was known for 703 male‐years in the data set and unknown for 51 male‐years. Female days held was not associated with *R*_GRM_ (*β *= −8.31E‐6 ± 1.24E‐5, *t*_1607_ = −0.672, *P* = 0.502X, adj. *R* ^2^ = −0.0003412). *R*_GRM_ was known for 8627 female–male pairs that did not mate, but where the female had been observed in the male's harem that particular year, in addition to 1446 pairs that did mate. Males with lower LBS were more closely related to the females in their harem (*β* = −6.065E‐4 ± 1.895E‐4, *t *= −3.201, *P* < 0.001), but within harems there was no difference in a male's relatedness to mated and nonmated females (*β* = −1.832E‐3 ± 3.563E‐3, *t* = −0.514). There was also no interaction between LBS and whether or not the pair mated (3.634E‐5 ± 1.763E‐4, *t* = −0.206), and thus no indication that males who sired more offspring avoided mating with related females within their harem.

## Discussion

### Selection on the act of inbreeding

This study presents a rare investigation of the strength of selection against the act of inbreeding, using data from a wild mammal population with strong polygyny and inbreeding depression, using both pedigree and genomic measures of relatedness.

We found that females do not vary in the degree to which they mate with relatives, using the genomic estimator of relatedness, and therefore that there is a lack of variation in this trait upon which selection can act. In addition, whereas there is a tendency for females that are more likely to mate with a relative to produce fewer calves, the strength of this association is very uncertain. There is therefore little expectation for females in this population to evolve kin avoidance or preference. When the fitness measure includes offspring survival, that is selection on the offspring being inbred, both pedigree and genomic measures indicate significant selection against inbreeding (LRS measures in Table 1; Fig. 3). This is consistent with previous research showing strong inbreeding depression in survival over the first 2 years of life (Walling *et al*., 2011; Huisman *et al*., 2016).

In contrast to females, for males the act of inbreeding was repeatable when estimated using both pedigree and genomic methods although the estimated repeatability was still low (~0.09 for both relatedness measures; Fig. 2). The repeatability is most likely caused by the strong philopatry of females, such that, if a male has one related female in his harem, he is likely to have several (Stopher *et al*., 2012). We found a negative association between genomic relatedness to mates and male fitness (measured as both LBS and LRS, Table 1, Fig. 3), indicating that males are under selection to avoid the act of inbreeding. However, based on the error around the estimated selection gradients (Fig. 3, Table 1), the strength of selection on the act of inbreeding did not differ significantly between the sexes, giving no support to the idea of a sexual conflict in selection on the act of inbreeding.

### Proximate explanations for selection against the act of inbreeding in males

Investigation of potential proximate mechanisms for the observed selection on males yielded only modest insights. As predicted, there were negative relationships between both male antler size and female days held and relatedness to mate, but they were not significant, which is perhaps not surprising given the sample sizes and low repeatability of relatedness to mate. Male age did predict *R*_GRM_ but in the opposite to expected direction, that is old males were more likely to mate relatives. This was due to rare father–daughter matings, and when these were removed, the relationship was also negative and not significant. Males that rut long enough to mate their daughters tend to be very successful, so it is interesting that we detected selection against relatedness to mate despite these cases. The fact that the *R*_GRM_ to actual mates did not differ from that of nonmated harem members shows that little discrimination against relatives or selection against inbred embryos is detectable with our data.

### Comparison of pedigree and genomic estimators

The point estimates for selection gradients on relatedness to mate and on an individuals’ own inbreeding coefficient were generally larger using the genomic compared with the pedigree estimators (Table 1). However, the standard errors were of similar magnitude, suggesting the difference is due to different parameter estimates rather than more precise estimates. Differences in the performance of pedigree and genomic estimators of inbreeding have been shown previously in this population. For example, inbreeding depression in adult fitness components was revealed using genomic estimates of inbreeding coefficients but not with pedigree inbreeding coefficients (Huisman *et al*., 2016).

These differences can be attributed to several factors. First, for males, sample sizes were considerably lower for pedigree data sets than for genomic data sets. This is because around 40% of breeding males are born elsewhere on the island to unknown parents, and for these *F*_PED_ and *R*_PED_ cannot be calculated. Consequently, the power to estimate selection is reduced for pedigree estimates. Second, the difference in performance between the two measures can be attributed to some degree to pedigree incompleteness among individuals for which both parents are known. In pedigree measures, an inbreeding coefficient or coefficient of relatedness to mates of zero has been assigned to a large proportion of individuals, whereas such a peak is not observed in the distributions of genomic measures (See Figs S1 and S2). This arises from a lack of information at deeper levels in the pedigree and unassigned paternity leading to the assumption that individuals are unrelated when in fact they are related to some degree. Finally, as outlined in the Introduction, genomic estimates of inbreeding and relatedness can capture variation around the expectation based on the pedigree measures that arise from sampling variance, Mendelian segregation and recombination (Visscher *et al*., 2006; Hill & Weir, 2011; Kardos *et al*., 2015; Huisman *et al*., 2016).

### Interpretation and implications

Our analyses indicate that, even in the presence of strong inbreeding depression, net selection against the act of inbreeding may not be strong. The lack of repeatability in relatedness to mate among females implies either that they do not or cannot choose between males on the basis of relatedness or that all females in the population follow the same strategy in the degree to which they mate with a relative. It is not possible to distinguish between these options with the current data, but whichever is true it suggests that female avoidance of kin is unlikely to evolve from its current state in this population.

The selection against mating with a relative among males but not females is in contrast to theoretical predictions that, under certain conditions, males should be more tolerant of inbreeding than females (see Introduction; (Waser *et al*., 1986; Kokko & Ots, 2006)) and to the empirical observation that males do hold female relatives in their harems and mate with them (Stopher *et al*., 2012). Our data suggest these males produce fewer offspring, and thus this strategy should be selected against. However, three points should be noted here: first, the repeatability of relatedness to a mate was still low in males, suggesting little variation among individuals in this trait, and this is reflected in the large uncertainty in the selection estimates (Fig. 3). Second, the heritability is unlikely to be larger than the repeatability (although see Dohm, 2002) and likely to be lower; thus, there may be limited scope for this trait to evolve. Third, it is important to consider the effect of males that do not gain any paternities and thus do not contribute to the estimate of the strength of selection against inbreeding in this population, the so‐called invisible fraction (Hadfield, 2009). As in many polygynous systems, male mating success is highly skewed in red deer – for example, among 455 SNP genotyped males that survived to at least 3 years old and held at least one female in a harem for at least 1 day, 43% sired zero offspring. These males could differ in their propensity to mate with a relative, but we cannot measure this variation. If these males have a particularly low propensity to mate with a relative, then our estimate of the strength of selection against inbreeding in males is likely to be a considerable overestimate. In addition, given the proportion of males that fail to produce any offspring in this population, it would appear to be more important for a male to gain any form of mating, rather than distinguishing between related or unrelated mates.

In both sexes, selection gradients were more negative and more often significant using the LRS measure compared with the LBS measure, that is when they incorporated offspring inbreeding depression (Table 1). Using this measure of fitness, it would pay individuals of both sexes to avoid inbreeding, although the caveat that males will be under even stronger selection to get any matings at all still applies. It is therefore worth considering the most likely routes by which each sex could avoid inbreeding. The strategies that philopatric female mammals deploy to avoid inbreeding have been reviewed by Clutton‐Brock (2016). Perhaps the simplest mechanism a female deer could use is to refuse to mate with a male that is familiar from her youth (who might be a maternal relative or paternal half‐sib) or, in the case of a young female, refuse to mate with a familiar rutting male (who might be her father). In both cases, this might manifest as females moving harems when approaching oestrus. Male red deer, which commonly disperse from their mothers around the age of 2 years to feed elsewhere, could avoid inbreeding by rutting away from their natal area. Both these possibilities are the subject of current investigation.

There are some additional caveats that should be borne in mind when interpreting the results of our study. First, when estimating the repeatability of relatedness to mate, we have tacitly assumed that the opportunities for an individual to mate with relatives and nonrelatives are similar from year to year. We think that this is reasonable because we nearly always observe opposite‐sex relatives and nonrelatives involved in the rut across the study area. Nevertheless, we suspect that the fact that relatedness to a mate is more repeatable in males than females is in part a consequence of the more clumped distribution of offspring sired over a male's lifetime than over female lifetimes, combined with the propensity of female relatives to be in the same harem. A male that holds a harem containing any relatives is likely to hold a harem containing multiple relatives, and the reproductive success gained from a single season of harem holding is likely to represent a considerable proportion of a male's lifetime offspring production. In contrast, females only produce a single offspring per year and produce offspring over multiple years, meaning any repeatability in female relatedness to a mate would have to occur across breeding seasons. Finally, we could only estimate relatedness to mate when a calf was born and sampled. Given that when observed intensively, females commonly only mate once or twice per oestrus and usually get pregnant in their first oestrus within a season, so do not cycle, we think this is a fair representation of mating behaviour. However, we do see some matings that do not lead to the birth of a calf (e.g. female mates with two males in the same oestrus, female cycles and conceives later, or female does not produce a calf at all) and so there is scope for an invisible fraction problem here as well.

To our knowledge, only one other study has estimated selection on the act of inbreeding, using social pairing data from a population of song sparrows on Mandarte Island, Canada (Reid *et al*., 2015a). In that study, in females, selection estimated at the earliest possible stage (number of banded offspring) *favours* mating with related males and remains positive but not significant when fitness is measured in terms of recruitment or grand‐offspring (i.e. in measures incorporating offspring inbreeding status). In males, there is no selection on relatedness to mate when fitness is measured in terms of banded offspring, but coefficients become negative (selection against the act of inbreeding) when fitness is measured at later stages and is significant for recruited grand‐offspring. Thus, in song sparrows, there is no selection against the act of inbreeding at any stage in females. In deer, our results indicate a lack of selection on relatedness to mate in female deer via LBS but selection against inbreeding when including offspring survival and in males using both measures of fitness. Empirical results from both studies therefore conflict with the hypothesis emerging from theory (Kokko & Ots, 2006; Puurtinen, 2011; Szulkin *et al*., 2013; Duthie *et al*., 2016; Duthie & Reid, 2016; Duthie *et al*., 2018) that in such systems females should be more strongly selected to avoid inbreeding than males. In both populations, as in many species, a large proportion of males produce no offspring and therefore selection against mating with a relative in males may be weak compared to selection to gain any matings at all, which is rarely considered in the theoretical models. Taken together, these results suggest that selection against the act of inbreeding may be weaker than expected from estimates of the magnitude of inbreeding depression alone (Duthie & Reid, 2016). Finally, in the deer, lifetime allelic fitness based on genomic data was highly correlated with LBS (see Methods), and in the song sparrows, pedigree LAF measures appear nearly identical to their LBS and LRS analogues (Reid *et al*., 2015a). Indirectly, this suggests that there is little potential for kin‐selected benefits of inbreeding in either population.

## Conclusions

In this study, we investigated whether there is evidence for selection against the act of inbreeding in a wild mammal population in which strong inbreeding depression is present. We found limited variation among individuals upon which selection could act, particularly in females. In terms of the total number of offspring produced, we found no evidence for selection against the act of inbreeding in females, but selection against inbreeding in males. However, this parameter was estimated with considerable uncertainty and, given the large fraction of males that never gain paternity, this selection may be trivial compared to selection to gain any matings at all. Thus, selection against the act of inbreeding may be weaker than expected from the observation of inbreeding depression. Finally, we could only detect this selection on the act of inbreeding when using genomic, rather than pedigree, estimates of relatedness and inbreeding. These results add to a number of recent studies suggesting that, even in populations with well‐resolved pedigrees, the additional information provided by genomic information at many loci can improve the power to detect effects of relatedness and inbreeding.

## Data Archiving

The data associated with the analyses presented are stored on Dryad, https://doi.org/10.5061/dryad.1b5f638

## Supporting information


**Figure S1** Distributions of relatedness to mate estimated from the pedigree (*R*_PED_) or directly from the SNPs using GCTA (*R*_GRM_).
**Figure S2** Distributions of inbreeding coefficients estimated from the pedigree (*F*_PED_) or directly from the SNPs using GCTA (*F*_GRM_) in females (left) and males (right).
**Figure S3** Distributions of lifetime breeding success (number of offspring born) and lifetime reproductive success (number of offspring surviving to independence) in females (left) and males (right).
**Figure S4** Predictions for tests of whether the relatedness of mated females differs from that of females in a male's harem and how this might vary in relation to male LBS.
**Figure S5** Relationship between the average relatedness to actual mates (y‐axes) and the average relatedness to all individuals of the opposing sex, weighted by the number of years they were both of reproductive age.
**Table S1** Mean, standard deviation (SD) and sample sizes (N) for numbers of offspring, measures of relatedness to mate (R), inbreeding (F) and fitness (lifetime breeding success (LBS) or lifetime reproductive success (LRS)) used in the analyses.
**Table S2** Repeatability of relatedness to mate.
