# Supplementary material for: Estimating selection on the act of inbreeding in a population with strong inbreeding depression
Source: J Evol Biol. 2018 Oct 16;31(12):1815–27. doi: 10.1111/jeb.13376 (PMC6334283; doi:10.1111/jeb.13376)
Supplement: Supplementary file 1 — Figure S1 Distributions of relatedness to mate estimated from the pedigree (R PED) or directly from the SNPs using GCTA (R GRM). Figure S2 Distributions of inbreeding coefficients estimated from the pedigree (F PED) or directly from the SNPs using GCTA (F GRM) in females (left) and males (right). Figure S3 Distributions of lifetime breeding success (number of offspring born) and lifetime reproductive success (number of offspring surviving to independence) in females (left) and males (right). Figure S4 Predictions for tests of whether the relatedness of mated females differs from that of females in a male's harem and how this might vary in relation to male LBS. Figure S5 Relationship between the average relatedness to actual mates (y‐axes) and the average relatedness to all individuals of the opposing sex, weighted by the number of years they were both of reproductive age. Table S1 Mean, standard deviation (SD) and sample sizes (N) for numbers of offspring, measures of relatedness to mate (R), inbreeding (F) and fitness (lifetime breeding success (LBS) or lifetime reproductive success (LRS)) used in the analyses. Table S2 Repeatability of relatedness to mate. [file JEB-31-1815-s001.docx]

**Supplementary Information**

*
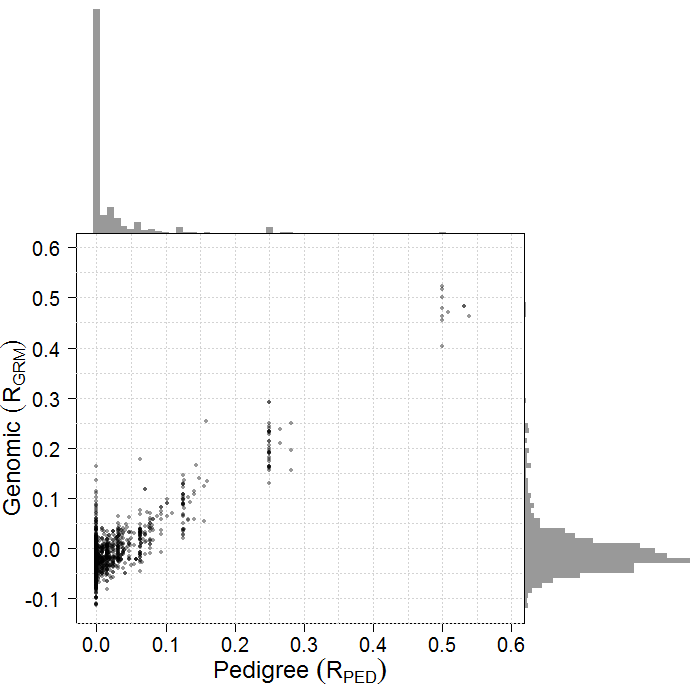
*

*Figure S1*: Distributions of relatedness to mate estimated from the pedigree (R_PED_) or directly from the SNPs using GCTA (R_GRM_). The figure shows all individuals for which both measures of relatedness to mate could be calculated.

| 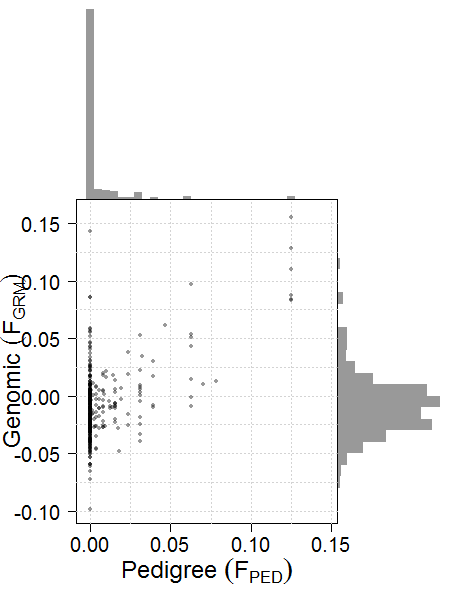 | 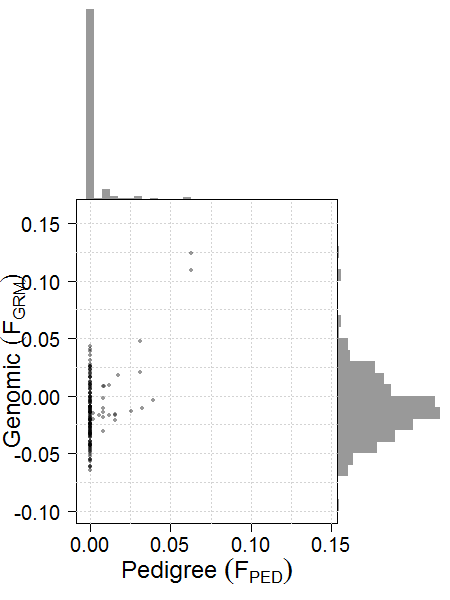 |
| --- | --- |

*Figure S2:* Distributions of inbreeding coefficients estimated from the pedigree (F_PED_) or directly from the SNPs using GCTA (F_GRM_) in females (left) and males (right). The scatterplot shows all individuals for which both measures could be calculated, while the histograms include all individuals for which each measure was calculated.

| **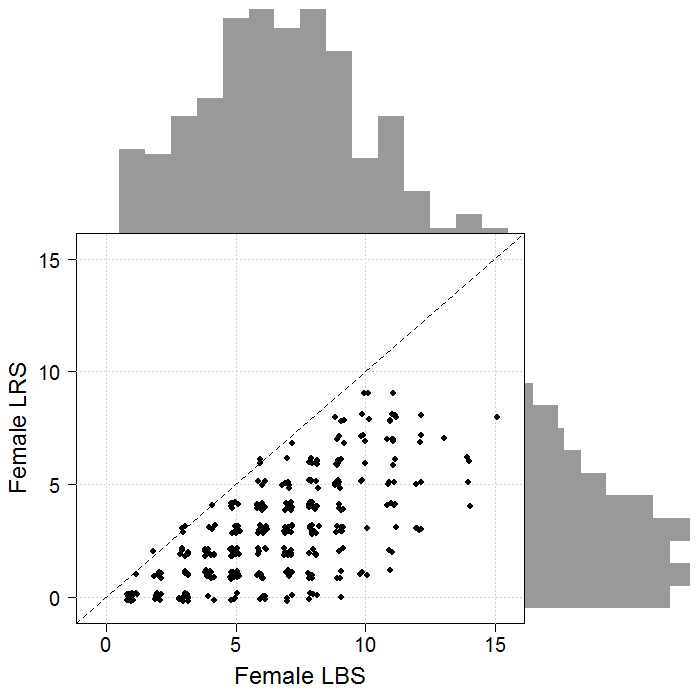** | **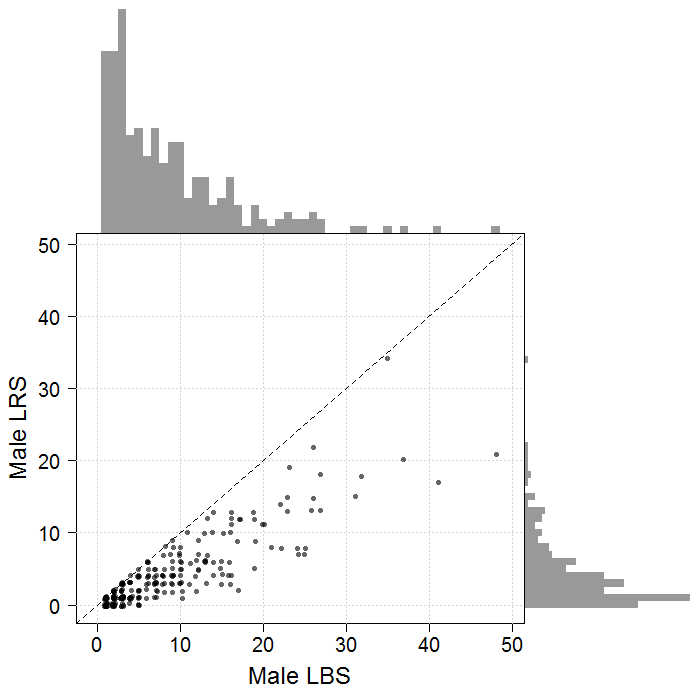** |
| --- | --- |

*Figure S2:* Distributions of lifetime breeding success (number of offspring born) and lifetime reproductive success (number of offspring surviving to independence) in females (left) and males (right). Note that only individuals that produced at least one calf are included in this dataset, but that calf did not have to survive to independence (i.e. minimum LBS = 1, but min LRS = 0). Points are jittered to illustrate density.


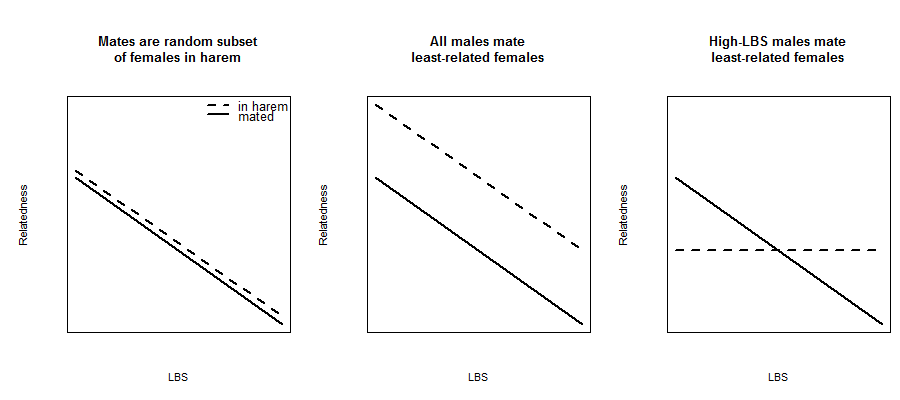


Figure S4 Predictions for tests of whether the relatedness of mated females differs from that of females in a male’s harem and how this might vary in relation to male LBS.

******

*Figure S5*: Relationship between the average relatedness to actual mates (y-axes) and the average relatedness to all individuals of the opposing sex, weighted by the number of years they were both of reproductive age. Lines show the one to one relationship, and the open point in the male dataset indicates the individual excluded in the outlier analysis. The extreme value in the female dataset produced a single inbred calf during her life.

Table S1: Mean, standard deviation (sd) and sample sizes (N) for numbers of offspring, measures of relatedness to mate (R), inbreeding (F) and fitness (lifetime breeding success (LBS) or lifetime reproductive success (LRS)) used in the analyses.

| **Measure** | **Sex** | **Relatedness and Inbreeding metric** | **mean** | **sd** | **N** |
| --- | --- | --- | --- | --- | --- |
| **Number of mates** | **Female** | **Pedigree** | **3.65** | **2.65** | **2394** |
| **Number of mates** | **Female** | **Genomic** | **3.43** | **2.40** | **1662** |
| **Number of mates** | **Male** | **Pedigree** | **6.92** | **7.30** | **2394** |
| **Number of mates** | **Male** | **Genomic** | **6.04** | **6.69** | **1662** |
| **Relatedness to mate (R)** | **Both** | **Pedigree** | **0.0190** | **0.0549** | **2394** |
| **Relatedness to mate (R)** | **Both** | **Genomic** | **-0.00206** | **0.0637** | **1662** |
| **Inbreeding (F)** | **Female** | **Pedigree** | **0.0054** | **0.0169** | **558** |
| **Inbreeding (F)** | **Female** | **Genomic** | **0.00856** | **0.0296** | **585** |
| **Inbreeding (F)** | **Male** | **Pedigree** | **0.00143** | **0.00537** | **200** |
| **Inbreeding (F)** | **Male** | **Genomic** | **-0.0172** | **0.0291** | **302** |
| **Fitness (LBS)** | **Female** | **NA** | **6.10** | **3.13** | **479** |
| **Fitness (LRS)** | **Female** | **NA** | **2.73** | **2.27** | **447** |
| **Fitness (LBS)** | **Male** | **NA** | **8.31** | **7.86** | **257** |
| **Fitness (LRS)** | **Males** | **NA** | **4.25** | **4.83** | **245** |

**Table S2: Repeatability of relatedness to mate**

| **Sex** | **Relatedness**  **metric** |  | **repeatability** | **SE** | **Chi^2^** | **P-value** |
| --- | --- | --- | --- | --- | --- | --- |
| Female | Pedigree |  | 0.0503 | 0.0168 | 12.2 | <0.001 |
|  |  |  |  |  |  |  |
|  | Genomic |  | 0.0322 | 0.0203 | 2.82 | 0.0929 |
|  |  |  |  |  |  |  |
| Male | Pedigree |  | 0.0875 | 0.0170 | 89.2 | <0.001 |
|  |  |  |  |  |  |  |
|  | Genomic |  | 0.0928 | 0.0213 | 62.8 | <0.001 |
|  |  |  |  |  |  |  |
